# Comparative analysis of colonization and survival strategies of regionally predominant LA-MRSA clones ST398 and ST9

**DOI:** 10.1128/msystems.00397-25

**Published:** 2025-09-09

**Authors:** Xing Ji, Yaxin Wang, Tao He, Henrike Krüger-Haker, Yang Wang, Congming Wu, Stefan Schwarz, Chengtao Sun

**Affiliations:** 1 National Key Laboratory of Veterinary Public Health and Safety, College of Veterinary Medicine, China Agricultural University 630101 , Beijing, China; 2 Jiangsu Key Laboratory for Food Quality and Safety—State Key Laboratory Cultivation Base of Ministry of Science and Technology, Institute of Food Safety and Nutrition, Jiangsu Academy of Agricultural Sciences 651111, Nanjing, China; 3 Institute of Microbiology and Epizootics, School of Veterinary Medicine, Center for Infection Medicine, Freie University Berlin 685237, Berlin, Germany; 4 Veterinary Center of Resistance Research, School of Veterinary Medicine, Freie University Berlin 9166, Berlin, Germany; Kobenhavns Universitet, Frederiksberg C, Denmark

**Keywords:** LA-MRSA, colonization, clonal dominance, pig, antimicrobial resistance

## Abstract

Livestock-associated methicillin-resistant *Staphylococcus aureus* (LA-MRSA) displays distinct geographical distribution patterns, with ST398 predominating in Europe and ST9 being the dominant lineage in Asia, particularly China. However, the mechanisms underlying these differences remain poorly understood. In this study, we evaluated the cell adhesion capacity, anti-phagocytic properties, and porcine nasal colonization potential of ST9 and ST398 strains isolated from China and Germany. Colonization dynamics and characteristics were further explored using 16S rRNA gene sequencing and metatranscriptomic analysis. Our findings revealed that LA-MRSA ST398 strains exhibited superior colonization capabilities, including enhanced cell adhesion, increased resistance to macrophage-mediated killing, and a stronger impact on nasal microbiota stability. Transcriptomic analyses during colonization indicated that ST398 strains prioritized pathways related to genome repair and amino acid metabolism, whereas ST9 strains, particularly those isolated from China, focused on carbohydrate metabolism. Although ST9 strains showed relatively weaker colonization capacity, the epidemic Chinese ST9 isolates carried multiple resistance genes [*fexA*, *tet*(L), and *aadE-spw-lsa*(E)-*lnu*(B)], exhibiting broad resistance to clinically important antibiotics including tylosin, florfenicol, and tetracyclines. This suggests that their prevalence in China may be maintained through antimicrobial selection pressure. With the implementation of stricter antibiotic use regulations in Chinese livestock production, ST398, due to its robust colonization potential, is likely to replace ST9 as the dominant LA-MRSA clone gradually.

**IMPORTANCE:**

Livestock-associated methicillin-resistant *Staphylococcus aureus* (LA-MRSA) is a significant public health concern due to its zoonotic potential and resistance to antimicrobial agents. Despite its global presence, the geographical dominance of specific clones, such as ST398 in Europe and ST9 in Asia, remains poorly understood. This study sheds light on the distinct colonization strategies and metabolic adaptations of these LA-MRSA lineages. By demonstrating the superior colonization abilities and metabolic versatility of ST398 compared to ST9, we speculate that changes in antimicrobial usage policies may drive a shift in the dominance of LA-MRSA clones in China’s livestock industry. These insights provide valuable guidance for managing LA-MRSA transmission and developing effective intervention strategies to mitigate its impact on animal and human health.

## INTRODUCTION

Livestock-associated methicillin-resistant *Staphylococcus aureus* (LA-MRSA) is a significant zoonotic pathogen, frequently causing widespread infections in animals and humans (1). Previous studies have demonstrated that LA-MRSA clonal lineages exhibit considerable diversity with distinct geographical distributions (2). For instance, ST398, a globally prevalent LA-MRSA lineage, has been detected in various animals and human populations across numerous European countries, North America, and South Korea (3). In contrast, the clonal lineage ST9 predominates in most Asian countries, particularly in China (4). However, the factors driving the differential prevalence of LA-MRSA ST398 and ST9 lineages in these regions remain unclear.

The emergence of dominant MRSA clones is shaped by dual selective pressures from the natural environment and host resistance. Studies have shown that the acquisition and loss of antimicrobial resistance and virulence genes play critical roles in the formation of prevalent clones, such as ST398 and ST9. For example, ST398 strains have acquired methicillin and tetracycline resistance genes while losing human-adaptive virulence factors, such as *sak*, *chp*, and *scn* (located on φSa3 prophages), facilitating their dominance in pigs (5). Similarly, the prevalence of ST9 strains is associated with their carriage of multiple antimicrobial resistance genes, enabling their persistence in the complex antimicrobial usage environment of farms in China (6). Although our previous research compared the genomic and *in vitro* fitness characteristics of MRSA ST398 and ST9 strains from China and Germany, it remains unclear whether differences in host colonization characteristics exist between these lineages. Furthermore, it is uncertain how such differences might influence their epidemiological distributions (7).

The nasal cavity is the primary site of *S. aureus* colonization in the host. To establish and persist in this niche, *S. aureus* must overcome several key challenges, including adhesion and invasion, immune evasion, and colonization resistance imposed by the host’s resident microbiota (8). Studies have shown that the expression of surface adhesion-related factors, such as iron-regulated surface protein A (IsdA), fibronectin-binding protein A (FnBPA), and clumping factor B (ClfB), facilitates persistent colonization and dissemination of *S. aureus* in the nasal cavity (9). In addition, *S. aureus* produces immune evasion proteins, including staphylococcal protein A (SpA), hemolysin-alpha (Hla), and Panton-Valentine leukocidin (LukSF-PV), to evade the host’s innate and adaptive immune defenses (10). The host’s nasal microbiota further influences *S. aureus* colonization by competing for attachment sites and nutrients, potentially inhibiting its colonization or enabling symbiotic coexistence. For example, the presence of *Finegoldia magna*, *Dolosigranulum pigrum*, and *Simonsiella spp*. has been negatively correlated with *S. aureus* colonization (11, 12). While few studies have specifically examined the colonization characteristics of MRSA ST9 and ST398, some reports indicate that MRSA/MSSA ST398 can establish stable colonization and spread within the nasal cavities of pigs, often co-colonizing with other *S. aureus* lineages (13). However, colonization by coagulase-negative staphylococci may counteract MRSA ST398 colonization over time (13). Despite these findings, current evidence is insufficient to fully explain the differential prevalence of MRSA ST9 and ST398 in China and Germany.

In this study, we investigated the adhesion capacity of MRSA ST9 and ST398 strains, isolated from China and Germany, to porcine nasal mucosal epithelial cells, as well as their resistance to macrophage-mediated phagocytosis. Furthermore, we established a porcine nasal colonization model and utilized 16S rRNA gene amplicon sequencing together with metatranscriptomic analysis to assess changes in the abundance of symbiotic microbiota and transcriptional activity of MRSA genes during nasal colonization. These analyses were conducted to elucidate the factors contributing to the differential prevalence of MRSA ST9 and ST398 strains, with particular emphasis on host colonization dynamics.

## MATERIALS AND METHODS

### Bacterial strains and culture

Based on our previous research, four methicillin-resistant *S. aureus* (MRSA) strains, classified as sequence types (STs) ST9 and ST398 and originating from China and Germany, were selected for this study (7). The four strains belonged to distinct evolutionary clusters and exhibited unique resistance phenotypes, with detailed isolation information and molecular genetic backgrounds provided in Table S1 and Fig. S1. The strains were cultured in a brain heart infusion medium at 37°C with shaking at 200 rpm overnight. Subsequently, 100 µL of the overnight bacterial culture was inoculated into 10 mL of fresh brain heart infusion (BHI) medium and incubated at 37°C for 4 hours to ensure logarithmic-phase growth. Before conducting *in vitro* and *in vivo* experiments, bacterial suspensions were adjusted to an appropriate density using sterile phosphate buffered saline (PBS).

### Porcine nasal epithelial cell adhesion assay

Immortalized porcine nasal mucosal epithelial cells were seeded in 24-well plates and cultured in epithelial cell medium (EpiCM) supplemented with 0.1% fetal bovine serum (FBS) to maintain ~90% confluency. Test bacteria were grown in BHI broth to the exponential phase, and the bacterial suspension was adjusted to 1 × 10^7^ CFU/mL. The epithelial cell monolayer was gently washed twice with 1× PBS before being inoculated with 500 µL of bacterial suspension at a multiplicity of infection (MOI) of 10:1. The plates were incubated at 37°C for 2 and 5 hours, respectively. After incubation, non-adherent bacteria were removed by washing the cell monolayers six times with antimicrobial-free EpiCM. To determine the total number of adherent and intracellular bacteria, cells were lysed with 500 µL of 0.5% Triton X-100, and the lysates were serially diluted. A 100 µL aliquot of each dilution was plated on brain heart infusion agar (BHA) and incubated at 37°C overnight for colony counting.

### Anti-phagocytosis assay

Mouse macrophage RAW 264.7 cells were seeded in 12-well plates and cultured in Dulbecco's modified Eagle medium (DMEM) containing 10% FBS until ~90% confluency. Exponentially growing bacteria were adjusted to ~3 × 10^7^ CFU/mL, and 500 µL of the bacterial suspension was added to the macrophages after rinsing with 1× PBS, with an infection rate (MOI) of approximately 5:1. The plates were incubated at 37°C for 1 hour. To eliminate extracellular and adherent bacteria, cells were washed twice with PBS and incubated with DMEM containing 200 µg/mL gentamicin for 40 minutes. Subsequently, cells were washed three times with PBS, lysed with 0.5% Triton X-100, and intracellular bacteria were released. Serial dilutions of the lysates (100 µL) were plated on BHA and incubated overnight at 37°C for colony counting. To assess intracellular bacterial viability, the infection period was extended to 3, 6, and 9 hours, and the same procedure was repeated as described above.

### Animal management and grouping

The animal study adhered to the Guidelines for the Care and Use of Laboratory Animals of the Chinese Association for Laboratory Animal Sciences and was approved by the Ethics Committee on Animal Experiments of China Agricultural University (AW82213202-2-4). The experiment was conducted in a barrier-level laboratory animal facility using 4-week-old, approximately 15 kg, weaned Landrace piglets from the same farm. Fifteen piglets were randomly divided into five groups for independent colonization assays, 16S rRNA gene sequencing, and metatranscriptome analysis, while 12 piglets were divided into four groups for the competition colonization tests. All piglets were confirmed to be healthy, MRSA-negative, and had not received antimicrobial therapy before the experiment. During the study, drinking water and feed were disinfected, and the piglets’ physiological status was monitored daily.

### Independent colonization assay and sampling

Piglets in four groups were inoculated intranasally with 1 mL of a 1 × 10^8^ CFU/mL suspension of CHN-/GER-MRSA ST9/ST398 in PBS using a dropper. Anterior nares samples were collected on days 1, 2, 3, 7, 14, and 21 post-inoculation using the ESwab system and processed for (i) bacteriological culture, (ii) MRSA quantification by qPCR, (iii) 16S rRNA microbiome sequencing, and (iv) metatranscriptome analysis.

For culturing, nasal swabs were enriched in 7.5% sodium chloride broth for 1 hour, followed by plating 100 µL of the culture on MRSA-selective media (with antimicrobials; see Table S2) and incubation at 37°C for 18–24 hours to enumerate colonies. The *mecA* gene levels were quantified by qPCR as a marker of MRSA colonization. DNA was extracted using the High Pure PCR Template Preparation Kit, with *gyrB* as the reference gene and CHN-ST9 as the control. Relative *mecA* expression was calculated using the ΔΔCt method (14).

### Competitive colonization ability

A 500 µL suspension containing 1 × 10^8^ CFU/mL of CHN- and GER-MRSA ST398 and ST9 was mixed in pairs (CHN MRSA ST9 vs CHN MRSA ST398, GER MRSA ST9 vs GER MRSA ST398, CHN MRSA ST9 vs GER MRSA ST398, and CHN MRSA ST398 vs GER MRSA ST398). The mixtures were then inoculated into the nasal cavities of piglets following the independent colonization assay protocol. Nasal swab samples were collected on days 1, 2, 3, 7, 14, and 21 post-inoculation for each group. The samples were enriched in 7.5% sodium chloride broth for 1 hour and subsequently plated on selective media designed to distinguish MRSA strains based on their specific antimicrobial resistance phenotypes (Table S2). Colony counts were performed to evaluate the competitive colonization ability of each strain pair.

### 16s rRNA sequencing and analysis

Total DNA from nasal swab samples was extracted using a modified SDS-based DNA extraction method. Amplicon libraries targeting the V3–V4 region of the 16S rRNA gene were prepared using universal primers 341F (5′-CCTAYGGGRBGCASCAG-3′) and 806R (5′-GGACTACNNGGGTATCTAAT-3′) (15). Sequencing was performed on an Illumina HiSeq System (PE250). Raw sequencing data underwent quality control with Trimmomatic v0.38, where low-quality reads (quality score < 20) and reads shorter than 80 nucleotides were removed. High-quality reads were assembled and denoised using USEARCH v11.2.64 and UNOISE to generate clean tags and zero-radius operational taxonomic units (ZOTUs). Species annotation of ZOTUs sequences was performed using the SINTAX algorithm (USEARCH v11.2.64) with the RDP 16S training set. Operational taxonomic units (OTUs) with a confidence level >0.8 were retained, and chloroplast- or mitochondria-related OTUs were excluded. Alpha diversity indices (e.g., richness, Chao1, and Shannon) were calculated using the USEARCH α_div subcommand, while beta-diversity distance matrices (e.g., weighted_unifrac, unweighted_unifrac, and Bray-Curtis) and principal coordinates analysis (PCoA) were computed using the USEARCH β_div subcommand (16). Differential species composition across groups at the phylum, class, order, family, genus, and species levels was analyzed using DESeq2 v1.26.0.

### Metatranscriptome sequencing

Nasal swab samples were transferred to 2 mL RNase-free tubes for RNA extraction using TRIzol reagent, with total RNA stored in nuclease-free water. RNA integrity and quantity were assessed using the Agilent 2100 Bioanalyzer and NanoDrop 1000 spectrophotometer. RNA-seq libraries were prepared with the Illumina RNA-seq Library Prep Kit, quality-checked on the Agilent 2100 Bioanalyzer, and sequenced on the Illumina NovaSeq platform. Raw sequencing data quality was evaluated using FastQC, and low-quality reads and adapters were removed with Trimmomatic v0.38. Ribosomal RNA sequences (28S, 18S, 5.8S, and 5S) were filtered out using SortMeRNA v4.2.0. Open reading frames were predicted from assembled transcriptomes using MetaProdigal v2.6.3. Functional annotation was performed with EggNOG v5.0, KEGG Kofam, and Cluster of Orthologous Groups (COG) databases, utilizing DIAMOND v2.1.3 and HMMER v3.1b2. KEGG orthology annotations were linked to KEGG categories such as Enzyme, Reaction, and Module. Functional abundance profiles at KEGG and COG levels were calculated using gene abundance information, reported as transcripts per million (TPM), and counts.

### Analysis of *in vivo* transcriptome expression of colonized MRSA

To analyze the *in vivo* transcriptome expression of colonized MRSA, we referenced a previous study and constructed a pan-genome library using nanopore whole-genome sequencing data from four MRSA strains obtained in this study (17). The pan-genome of *S. aureus*, comprising core and accessory components, was constructed from the four genomes and included 2,761 orthologous groups (OGs) identified via BLASTp. RNA-seq transcript reads were mapped to *Staphylococcus* spp. Using this database, metatranscriptomic data sets, with pig-derived and ribosomal reads removed, were individually analyzed using the custom BLAST database. Reads with an alignment identity of ≥90% were aggregated and assigned as originating from *S. aureus*. Functional annotation of all OGs was performed using the EggNOG v5.0, KEGG Kofam, and COG databases as described previously.

### Statistical analysis and visualization

Differential statistical analyses of grouped data were performed using the Kruskal-Wallis test, Mann-Whitney *U* test, or Student’s *t*-test, with statistical significance set at *P* < 0.05. Analyses were conducted using GraphPad Prism v8.0.2. Microbiome α-diversity (Chao1 index) and β-diversity (PCoA and Bray-Curtis distances) were calculated based on normalized relative abundance data using the vegan package in R. For transcriptomic data visualization, differential COGs and KEGG pathways were illustrated using butterfly plots to highlight upregulated and downregulated pathways. Expression distribution patterns across MRSA strains were displayed using ridge density plots, while pathway expression profiles were represented as heatmap bubble plots. All visualizations were generated with R packages, including ggplot2, ggridges, and pheatmap, employing customized color palettes and clustering parameters to enhance clarity and interpretability.

## RESULTS

### MRSA ST398 exhibited stronger adhesion and anti-phagocytic ability than ST9

**Fig 1:**
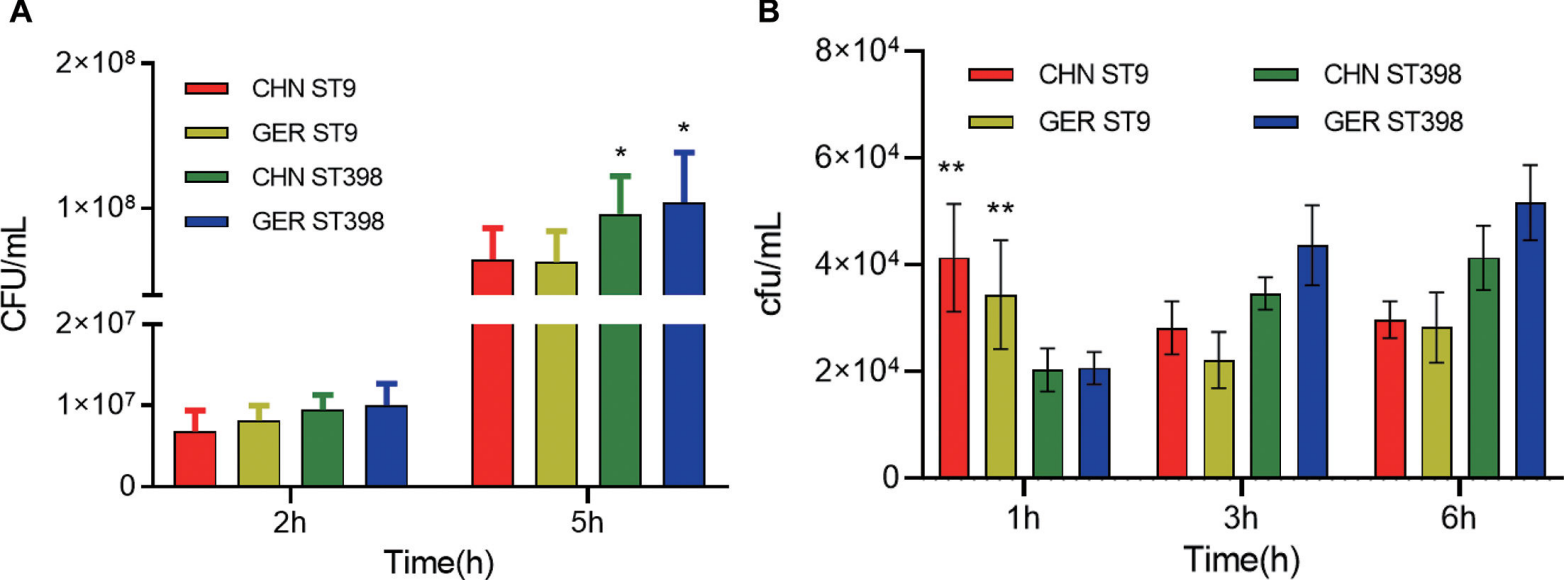
Adhesion ability to porcine nasal epithelial cells and anti-macrophage killing ability of MRSA ST398 and ST9 strains. (**A**) Intracellular bacterial load in nasal mucosal epithelial cells at 2 and 5 hours. (**B**) Comparison of the anti-phagocytosis and anti-macrophage killing abilities of MRSA ST398 and ST9 strains at 1, 3, and 6 hours, expressed as intracellular bacterial load.

We employed immortalized porcine nasal epithelial cell lines and the mouse macrophage cell line RAW264.7 to assess the adhesion and phagocytosis resistance of MRSA ST9 and ST398 strains isolated from Germany and China. Following a 2-hour co-incubation of epithelial cells with the bacteria, no significant difference in intracellular bacterial load was observed between the ST9 and ST398 strains. However, by the fifth hour, the intracellular bacterial load of MRSA ST398 in epithelial cells was significantly higher than that of MRSA ST9 (*P* < 0.05, ANOVA; Fig. 1A), suggesting enhanced persistence in colonization of the ST398 strains. In addition, in macrophage interaction studies, we found that the intracellular bacterial load of MRSA ST398 strains progressively increased over time, whereas ST9 exhibited a declining trend. Notably, MRSA ST398 isolates displayed a lower intracellular bacterial load at 1 hour compared to ST9, suggesting enhanced resistance to phagocytosis (*P* < 0.05, ANOVA). Over time, ST398 strains demonstrated significantly higher intracellular loads at 3 and 6 hours, indicating superior resistance to macrophage-mediated killing relative to ST9 (Fig. 1B).

### MRSA ST9 and ST398 revealed different capacities of colonization and competitive colonization

**Fig 2:**
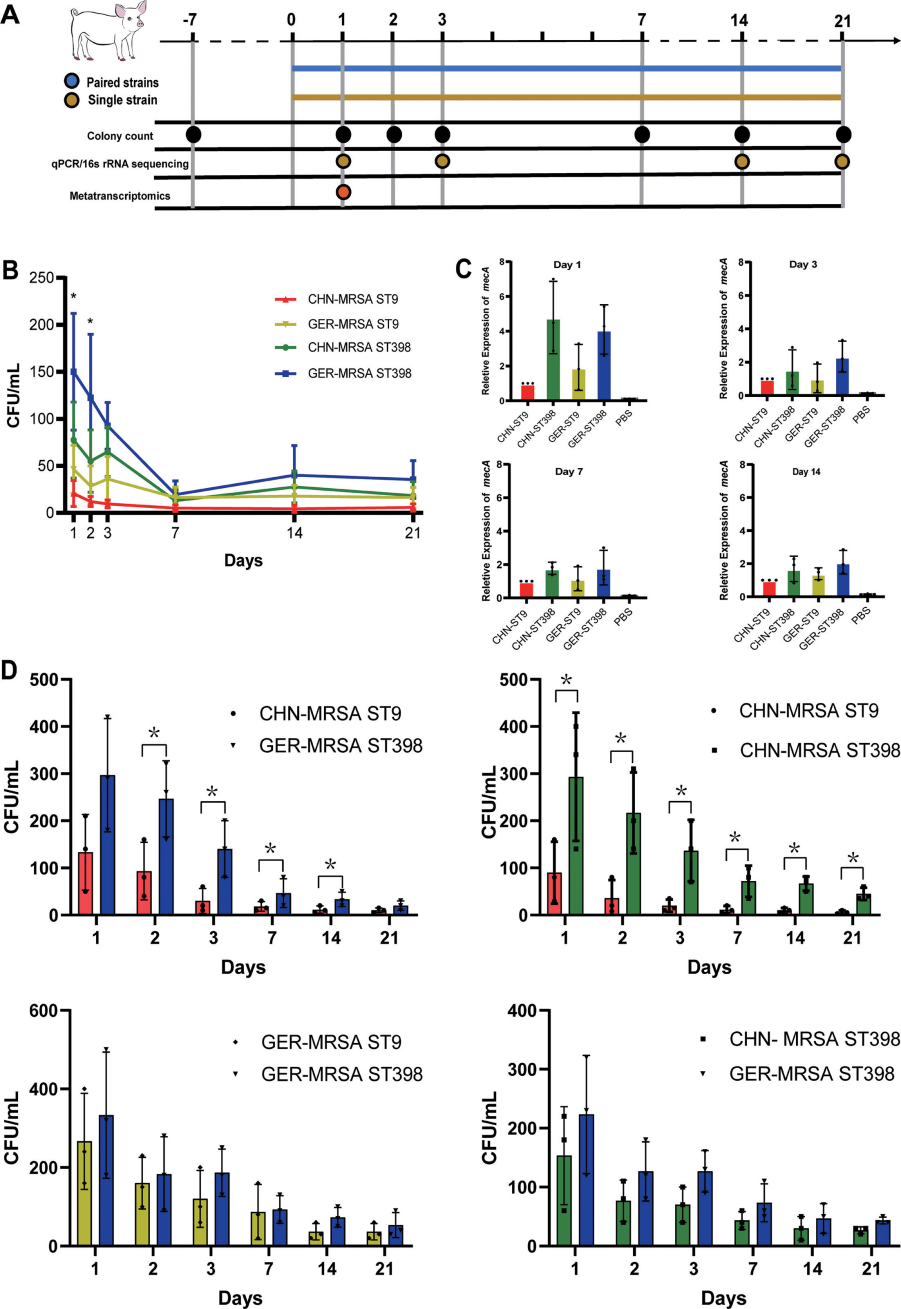
Colonization ability and competitive colonization of MRSA ST9 and ST398 in swine nasal cavities. (**A**) Sampling process of swine nasal colonization assay. (**B**) The colonization ability of different strains in the pig nasal cavity over 21 days. (**C**) The relative abundance of the *mecA* gene represents the colonization ability of different MRSA strains. (**D**) The competitive colonization of each paired strain was detected using the colony counting method.

The experimental design for host colonization testing of MRSA ST9 and ST398 strains is illustrated in Fig. 2A. Seven days prior to colonization, all piglets were confirmed to be free of *S. aureus* (including MRSA) in their nasal cavities by sampling and culturing on *S. aureus* chromogenic medium. In the independent colonization assay, the colonization levels of all tested strains showed a decreasing trend that eventually stabilized within 21 days. On days 1 and 2, the colonization levels of both CHN- and GER-MRSA ST398 strains were significantly higher than those of MRSA ST9 strains (*P* < 0.05, *t*-test), with CHN-MRSA ST9 being nearly undetectable by day 21. Overall, GER- and CHN-MRSA ST398 strains demonstrated superior colonization capacity and persistence (Fig. 2B). In addition, we used the relative quantitative PCR results of *mecA* gene abundance to calibrate the colony counting method and received similar results (Fig. 2C).

To further assess colonization competitiveness in the pig nasal cavity, co-colonization assays were conducted. Consistent with the independent colonization results, both CHN- and GER-MRSA ST398 strains exhibited significantly higher colonization competitiveness compared to MRSA ST9 strains from the same regions, with this competitive advantage persisting through day 21. Notably, no significant differences were observed in colonization competition between CHN- and GER-MRSA ST398 strains. However, CHN-MRSA ST9 displayed the weakest colonization competitiveness overall (Fig. 2D).

### MRSA ST9 and ST398 colonization affect the composition of the nasal microbiota

**Fig 3:**
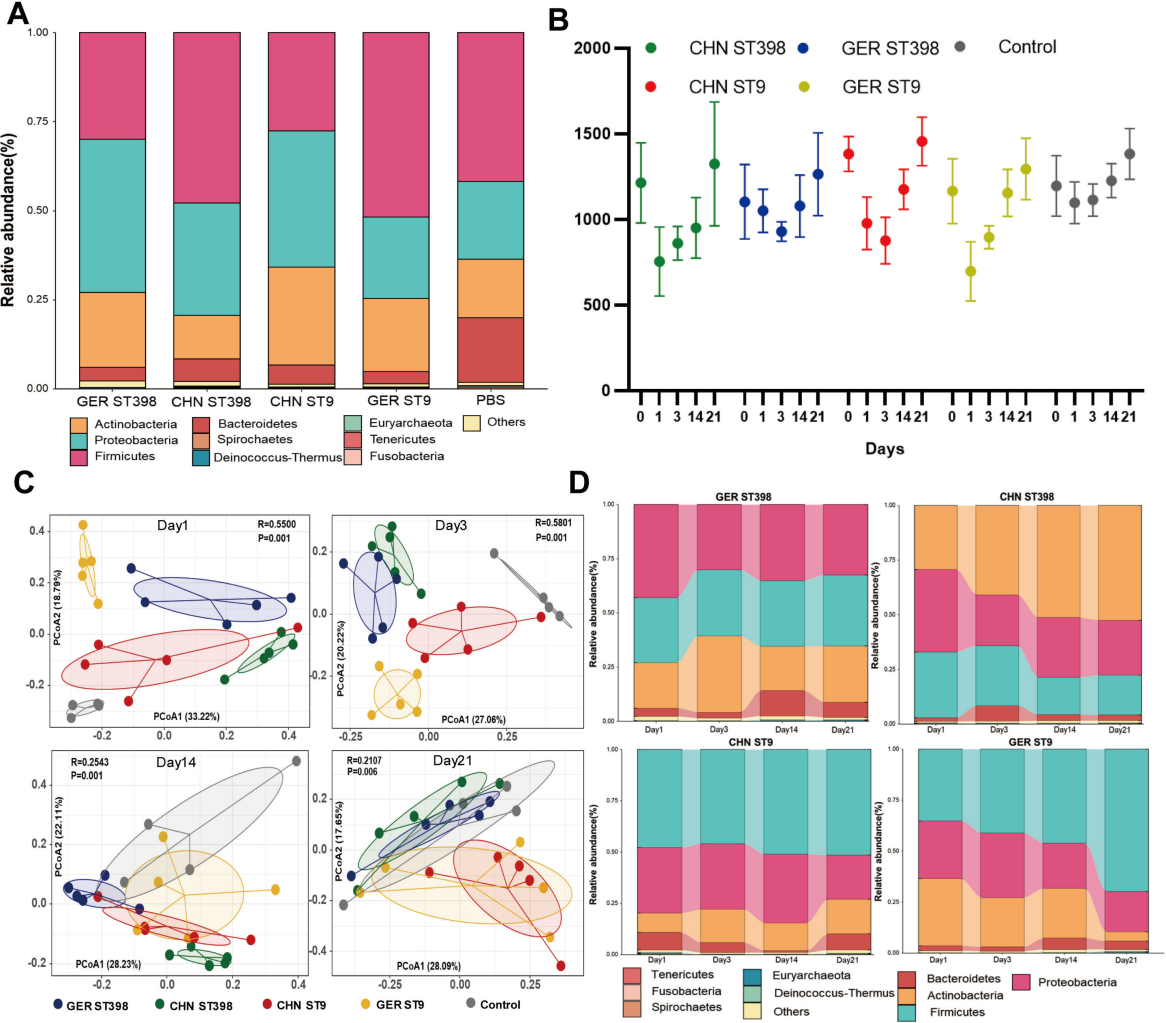
Effects of colonization with MRSA ST9 and ST398 strains on the porcine nasal microbiota. (**A**) Phylum-level composition of pig nasal microbiota before MRSA ST9 and ST398 strains colonization. (**B**) Alpha diversity analysis of samples from each group based on the Chao1 index on days 1, 3, 14, and 21 after colonization. (**C**) The PCoA based on Bray-Curtis distances reflects the differences in microbiota composition among samples from different groups. (**D**) Changes in phylum levels of porcine nasal microbiota after 1, 3, 14, and 21 days of colonization with MRSA ST9 and ST398 strains. The relative abundance of bacterial communities was quantified using TPM.

Before the MRSA ST9/ST398 colonization experiment, α-diversity analysis of nasal samples revealed no significant differences in microbiota composition or abundance among the experimental groups (Table S3), aligning with the study’s expectations. Taxonomic classification of OTUs at the phylum level showed that the nasal microbiota composition were highly similar across all groups. The dominant phyla included *Firmicutes* (35%–40%), *Proteobacteria* (25%–35%), *Actinobacteria* (15%–35%), *Bacteroidetes* (1%–5%), and others (Fig. 3A). At the genus level, the predominant genera were *Rothia* (15%–35%), *Moraxella* (10%–30%), *Streptococcus* (5%–18%), *Lactobacillus* (5%–15%), *Prevotella* (1%–10%), *Chryseobacterium* (1%–5%), *Clostridium* (1%–3%), *Blautia* (1%–2%), and *Collinsella* (1%–1.5%; Fig. S2).

Following inoculation with MRSA ST9 or ST398 strains, nasal samples were subjected to longitudinal 16S rRNA sequencing at 1, 3, 14, and 21 days post-colonization. Alpha diversity analysis, based on the Chao1 index, revealed a decline in bacterial community diversity initially, followed by an increase, indicating that MRSA colonization significantly disrupted microbial diversity (Fig. 3B). Beta diversity ANOSIM analysis showed significant differences in microbial community composition between MRSA-colonized groups and the control group across all time points (*R* > 0, *P* < 0.05; Fig. S3). PCoA based on Bray-Curtis distances demonstrated clustering within the same colonization group compared to the control group. Over time, the inter-sample distances within each group decreased, indicating that the microbiota became more consistent as colonization progressed (Fig. 3C).

### Changes in the abundance of *Firmicutes*, *Bacteroidetes*, and *Proteobacteria* are associated with the colonization of MRSA ST9 and ST398

OTU analysis revealed that colonization with MRSA ST9 or ST398 strains had minimal impact on the composition and diversity of the dominant phyla within the nasal microbiota. However, over time, the abundance of *Firmicutes*, *Bacteroidetes*, and *Proteobacteria* varied across the different colonization groups (Fig. 3D). To identify specific genera associated with MRSA ST9 and ST398 colonization, we utilized DESeq2 to compare nasal microbiota changes between colonization groups (Fig. S4). Compared to CHN- and GER-MRSA ST9 colonization groups, CHN- and GER-MRSA ST398 groups exhibited a relative increase in the abundance of *Actinobacteria* and *Proteobacteria*, alongside a decrease in *Firmicutes*. At the genus level, the abundance of *Acinetobacter*, *Sphingomonas*, *Pseudomonas*, *Comamonas*, *Aeromonas*, and *Corynebacterium* increased, while *Faecalibacterium*, *Weissella*, and *Peptococcus* decreased (Table S4). These genus-level differences in abundance were consistently observed across the two sampling points over the 21 days.

Furthermore, metatranscriptomic analysis from the first day post-colonization confirmed that *Actinobacteria* and *Proteobacteria* exhibited higher transcriptional activity in GER-MRSA ST398 groups, whereas *Firmicutes* were more transcriptionally active in CHN-MRSA ST9 groups during the early colonization stage (Fig. S5).

### MRSA ST398 and ST9 strains exhibit limited and similar effects on the transcriptional activity of the microbiota

**Fig 4:**
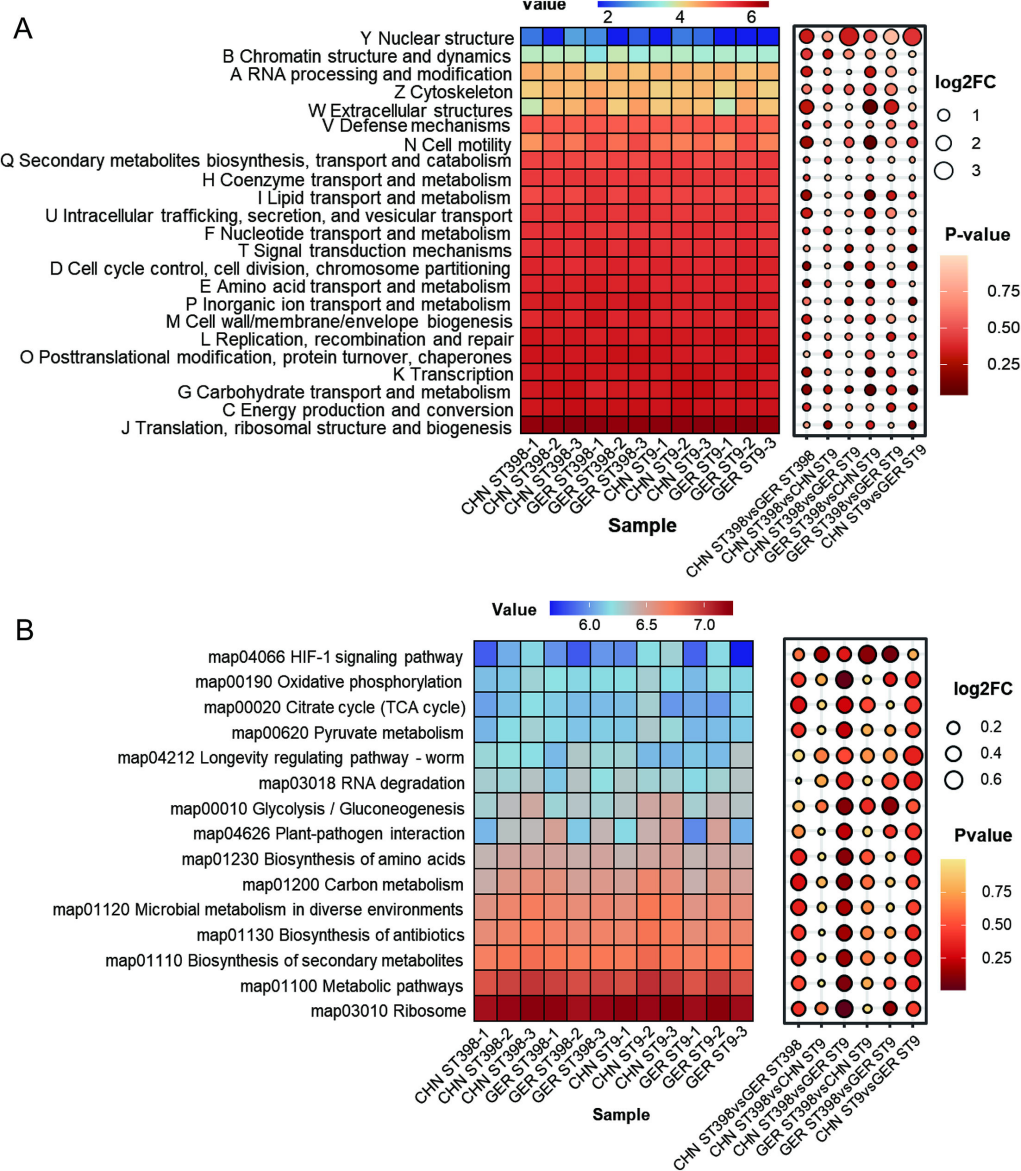
Gene expression characteristics of total nasal microbiota in different colonization groups based on COGs and KEGG and differential analysis of gene expression in different groups. (**A**) Analysis of functional gene expression based on COG classification (heatmap) and differential expression of COG functional genes between two groups (bubble plot). (**B**) Analysis of functional gene expression based on level 3 pathways (heatmap) and differential expression of functional genes between two groups (bubble plot).

To determine whether MRSA ST398 and ST9 colonizations influence the functional and metabolic gene expression of the porcine nasal microbiota, we performed metatranscriptomic sequencing and annotated the functional genes using the COG and KEGG databases. COG annotation revealed that genes related to category J (translation, ribosomal structure, and biogenesis) were the most actively expressed across all MRSA colonization group samples, while the expression levels of other functional categories were relatively low (Fig. 4A). Similarly, KEGG pathway (level 3) analysis showed that genes associated with the ribosome (map03010) and metabolic pathways (map01100) were highly expressed in all samples. These findings suggest that protein synthesis and metabolic activities within the nasal microbiota were significantly upregulated during MRSA colonization, likely reflecting a rapid proliferation and metabolic response to counteract the initial colonization by MRSA (Fig. 4B).

Notably, no differentially expressed functional categories were observed between the MRSA ST398 and ST9 colonization groups at either the COG or KEGG level 3 pathway level (log2FC < 1 and *P* > 0.05). Furthermore, Bray-Curtis distance-based PCoA demonstrated that the abundance and composition of functional and metabolic genes were highly similar among all samples, with no significant clustering based on geographic origin or ST (Fig. S6). These results indicate that MRSA ST9 and ST398 colonizations have a minimal impact on the transcriptional activity of metabolic and other functional genes in the nasal microbiota of pigs.

### ST9 prefers to activate the features of carbon source metabolism, while ST398 prefers to reorganize and repair the genome

**Fig 5:**
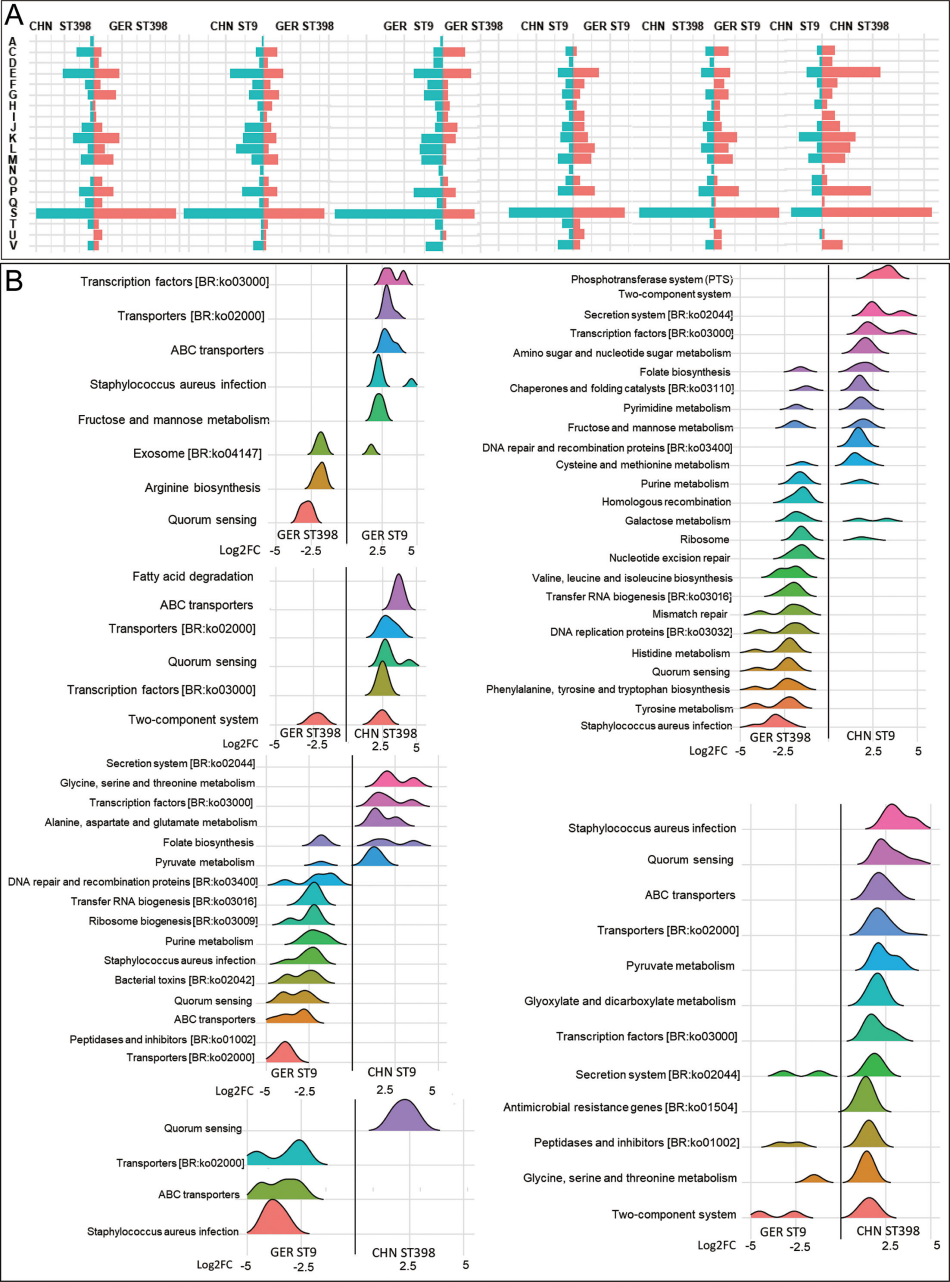
Gene expression characteristics and differential gene expression analysis of colonizing strains MRSA ST398 and ST9 based on COG and KEGG level 3 pathway annotations. (**A**) Relative abundance analysis of functional gene expression was performed according to the COG classification. (**B**) Distribution of genes with significant differential expression between MRSA ST9 and ST398 strains as a ridge plot (log2FC > 2, *P* < 0.05). A, RNA processing and modification; C, Energy production and conversion; D, cell cycle control, cell division, and chromosome partitioning; E, amino acid transport and metabolism; F, nucleotide transport and metabolism; G, carbohydrate transport and metabolism; H, coenzyme transport and metabolism; I, lipid transport and metabolism; J, translation, ribosomal structure, and biogenesis; K, transcription; L, replication, recombination, and repair; M, cell wall/membrane/envelope biogenesis; N, cell motility; O, posttranslational modification, protein turnover, and chaperones; P, inorganic ion transport and metabolism; Q, secondary metabolites biosynthesis, transport, and catabolism; S, function unknown; T, signal transduction mechanisms; U, intracellular trafficking, secretion, and vesicular transport; V, defense mechanisms.

To explore the transcriptional characteristics of MRSA ST9 and ST398 strains during colonization in pig nasal cavities, sequenced reads from each sample were mapped to their respective whole genome reference sequences. Differential gene expression analysis was performed using stringent criteria (Benjamini-Hochberg adjusted *P*-value < 0.05 and log₂ fold change ≥ 2). The results indicated that the CHN-ST9 and GER-ST398 paired groups exhibited the highest number of significantly differentially expressed genes, followed by comparisons between CHN-ST9 and GER-ST9 strains. COGs annotation revealed fewer differentially expressed functional genes between CHN and GER ST398 strains, primarily in categories related to cellular processes and signal transduction (categories D and O). In contrast, CHN and GER ST9 strains displayed more differentially expressed genes across categories related to information storage and processing, cellular processes and signaling, and metabolism (categories C, E, I, L, and U). Comparisons between different sequence types (e.g., ST398 and ST9 strains from China or Germany) identified more differentially expressed genes within information storage, processing, and metabolism-related categories, such as C, E, J, K, L, P, and V (Fig. 5A).

KEGG pathway analysis supported these findings (Fig. 5B), showing fewer differentially expressed genes between CHN and GER ST398 strains, mainly within pathways related to fatty acid degradation, transport proteins, and quorum sensing. In contrast, CHN and GER ST9 strains demonstrated expression differences across a broader range of metabolic and biosynthetic pathways, including amino acid metabolism, pyruvate metabolism, and ribosome biogenesis. Notably, CHN and GER ST398 strains exhibited fewer differentially expressed functional genes compared to GER ST9 strains but more when compared to CHN ST9 strains. Specifically, CHN and GER ST398 strains showed higher expression of genes related to DNA repair and replication, amino acid metabolism, and quorum sensing (e.g., homologous recombination and histidine metabolism pathways). Conversely, ST9 strains demonstrated higher expression of genes associated with carbohydrate metabolism and signal transduction, including the phosphotransferase system and two-component system pathways.

In summary, both COG and KEGG analyses suggest that CHN and GER ST398 strains share similar colonization and gene expression profiles in pig nasal cavities, whereas significant transcriptional differences exist between CHN and GER ST9 strains.

### High expression of genes related to metabolic adaptability and genome stability may contribute to the colonization and prevalence of MRSA ST398 and ST9

Our previous findings revealed that CHN- and GER-MRSA ST398 strains exhibit similar transcriptional profiles and superior colonization ability compared to ST9 strains, particularly CHN ST9. To further investigate the mechanisms underlying the widespread prevalence of GER ST398 and CHN ST9 strains, as well as the enhanced colonization capacity of CHN and GER ST398, we performed an in-depth analysis of highly expressed genes in CHN/GER ST398 and CHN ST9 strains during the colonization phase.

Compared to the ST9 strains, genes commonly upregulated in both CHN and GER ST398 strains were primarily associated with two functional categories: E (amino acid transport and metabolism) and P (inorganic ion transport and metabolism). These upregulated genes encoded key proteins such as the ABC transporter substrate-binding protein (*appA*), D-lactate dehydrogenase (*fdh*), anthranilate synthase component 1 (*trpE*), phosphate import ATP-binding protein (*pstB*), oligopeptide transport ATP-binding protein (*oppD*), and L-threonine dehydratase biosynthetic (*ilvA*). In addition, the GER ST398 strain uniquely exhibited high expression of genes in category L (replication, recombination, and repair), including those encoding DNA polymerase beta (*polX*), methylated-DNA–protein-cysteine methyltransferase (*ogt*), and transcriptional repressor (*ccpN*).

In contrast, the prevalent CHN ST9 strain displayed higher expression of genes within category G (carbohydrate transport and metabolism) compared to the GER ST9 strain. These included mannose-6-phosphate isomerase (*manA*), phosphoglycerate mutase (*gpmA*), glycerate kinase (*glxK*), and dihydropteroate synthase (*folP*). These findings suggest that the elevated expression of genes associated with metabolic adaptability and genomic stability plays a pivotal role in the adaptive colonization of porcine nasal cavities and the widespread dissemination of dominant MRSA ST398 and ST9 strains.

## DISCUSSION

To the best of our knowledge, this is the first study to comprehensively compare the adhesion capacity to porcine nasal epithelial cells, resistance to phagocytosis, colonization capacity in the porcine nasal cavity, interactions with nasal microbiota, and functional gene expression changes during the colonization process of MRSA ST9 and ST398 strains from both China and Germany. Our findings offer valuable insights into the colonization dynamics and mechanisms underlying the dominance of LA-MRSA clones.

The nasal cavity in animals and humans serves as the primary niche for *S. aureus* colonization, and the capacity for host colonization is a critical factor influencing strain prevalence (18). Our findings revealed that both CHN and GER MRSA-ST398 strains exhibited more persistent and competitive colonization compared to MRSA-ST9 strains, particularly CHN ST9, in both *in vitro* and *in vivo* models. This difference became increasingly pronounced over time. These results suggest that ST398 strains possess a greater capacity for colonization in porcine nasal tissue, aligning with observations reported in previous studies (19, 20). Furthermore, MRSA ST398 strains demonstrated enhanced resistance to macrophage phagocytosis, indicating their superior ability to evade the host’s innate immune response. Recent studies also suggest that MRSA ST398 clonal strains are more likely to cause systemic infections in the host (21–23). Consistent with previous studies, the predominant microbial genera in the pig nasal cavity before MRSA colonization included *Rothia*, *Moraxella*, *Staphylococcus*, and *Lactobacillus*. These findings suggest that the composition of the pig nasal microbiota is minimally influenced by geographic location or varying feeding conditions (24). Colonization with MRSA ST398 and ST9 strains temporarily disrupts the composition of the nasal microbiota; however, the microbiota composition gradually recovers over the course of the colonization process. This suggests that the natural nasal microbiota has inherent resistance to MRSA colonization (25). Although the colonization of MRSA ST398 and ST9 did not significantly affect the overall composition and diversity of the microbiota, some differences in the abundance of specific bacterial taxa were observed. Both CHN and GER ST398 strains had a more substantial impact on the abundance and activity of *Actinobacteria* or *Proteobacteria*, suggesting that ST398 strains are more likely to influence nasal microbiota stability compared to ST9 strains. This may potentially facilitate the proliferation of some pathogenic gram-negative bacteria, thereby increasing the risk of host infection. For instance, the increase in *Proteobacteria* has been associated with airway hyperresponsiveness (26). It is noteworthy that *Lactobacillus* and *Weissella* had higher abundance and activity in the CHN-ST9 colonization group. It has been reported that strains of *Lactobacillus* and *Weissella* can inhibit the growth of *S. aureus* or MRSA isolates *in vitro* by producing acid or bacteriocin-like inhibitors (27–29). Our previous findings indicated that CHN-MRSA-ST9 exhibits reduced acid tolerance, potentially elucidating its unexpectedly lower *in vivo* colonization capacity compared to GER-MRSA-ST9, despite their similarities *in vitro* adhesion and invasion properties (7). These results indicate that the colonization environment in animals is more complex, and bacteria must cope with various physical stresses. *In vitro*, epithelial cell adhesion assays alone cannot fully simulate the colonization conditions in the host (30).

Nasal environmental secretions not only lack monomeric nutrients and energy sources but also have low levels of essential ions such as trace metals and phosphate (31). Metatranscriptomic analysis revealed that while MRSA ST398 and ST9 strains did not significantly affect the functional gene expression of the commensal microbiota during colonization, the strains themselves exhibited distinct gene expression patterns throughout the colonization process. This differential gene expression may help explain the discrepancy in their epidemiological transmission and colonization abilities. Notably, ST398 strains from both China and Germany demonstrated elevated expression of genes associated with amino acid transport and metabolism (COG category E) and inorganic ion transport and metabolism (COG category P). This gene expression profile suggests that ST398 possesses superior nutrient acquisition and metabolic adaptation, potentially conferring a competitive advantage in nutrient-limited environments (32, 33). For example, the highly expressed *pstB* gene, encoding an ATP-binding protein of the PstSCAB phosphate transport system, can enhance the ability of MRSA ST398 to acquire phosphate and nitric oxide resistance, thereby improving its growth rate and reproductive capacity (34). In addition, *oppD* is part of the typical Opp transport system, which can transport various oligopeptides. The high expression of *oppD* can help the strain to use more free oligopeptides as carbon sources (35). The high expression of replication, recombination, and repair (COG L) genes suggests that ST398 may have stronger genome stability and adaptability, which helps it maintain genetic integrity in the host environment. For example, under oxidative stress conditions, the high expression of *polX* may help the strain mitigate oxidative stress and repair DNA damage (36). MRSA ST9, especially the CHN ST9 strain, had a higher expression of carbohydrate (COGs G)-related genes, which is consistent with our previous findings that the Chinese MRSA ST9 strain exhibits slower amino acid metabolism but increased carbohydrate metabolism (37). These results indicate that different MRSA clones have adopted distinct survival strategies during evolution. ST398 appears to focus on diversified nutrient acquisition (amino acids, peptides, and inorganic ions), while ST9 prioritizes carbohydrate utilization. This may explain why different MRSA clones have a limited effect on the abundance of specific commensal bacterial genera, though the precise mechanisms require further investigation.

Our findings suggest that CHN-MRSA ST9, as a prevalent strain, exhibits weak colonization ability, which may seem counterintuitive. However, it is important to note that our experiments were conducted without antimicrobial selection pressure. MRSA ST9 isolated from China carries multiple antimicrobial resistance genes, such as the *fexA*, *tet*(K), *tet*(M), and *aadE-spw-lsa*(E)*-lnu*(B) gene clusters, potentially incurring a substantial fitness cost (7, 38). Prior to the 2020 prohibition on antibiotic growth promoters in China, Chinese livestock farms extensively utilized antimicrobial agents, including amoxicillin, florfenicol, tetracycline, and tylosin as growth promoters (39). This practice exerted substantial selective pressure that likely contributed to the emergence and dissemination of multidrug-resistant MRSA ST9 clones. We hypothesize that CHN-MRSA ST9 will demonstrate a strong competitive advantage in intensive farming with high levels of antimicrobial use while offsetting the fitness cost of antimicrobial resistance genes through enhanced carbohydrate metabolism. In comparison, CHN-MRSA-ST398 exhibits superior colonization ability and *in vivo* persistence. As China implements restrictions and reduces antimicrobial use in livestock farming, CHN-MRSA ST398 may gradually replace ST9 as the predominant clone. Indeed, recent reports indicate an increasing prevalence of MRSA ST398 isolates in China (23, 40). The potential widespread dissemination of MRSA ST398 in China could pose substantial public health challenges. ST398 possesses significantly greater cross-species transmission capacity and enhanced virulence potential relative to ST9, which may increase the risk of spread from farms to communities and medical institutions and may cause more severe infections in animals and humans (41). Therefore, it is necessary to continue strengthening dynamic monitoring of the molecular evolution of ST398.

However, there are certain limitations in this study, such as the limited number of MRSA ST398 and ST9 test strains and the lack of *in vitro* transcriptome sequencing analysis based on cell adhesion experiments. Nevertheless, our findings highlight the complex interactions among host immunity, microbiota, and the physicochemical environment in MRSA colonization and transmission. They also underscore the importance of metabolic flexibility and genome repair capability in the colonization and transmission of prevalent MRSA clones. For LA-MRSA predominant clones (such as ST398 or ST9), future research should focus on comparative phenotypic analyses across host species (swine, poultry, and humans) to elucidate niche-specific adaptations, alongside developing targeted interventions, such as phage therapy, probiotics, and improved farm biosecurity measures to complement antibiotic stewardship programs.

## Data Availability

The 16S rRNA gene sequencing and metatranscriptome datasets generated in this study have been deposited in the Sequence Read Archive (SRA) database of the National Center for Biotechnology Information (NCBI) under the BioProject accession numbers PRJNA1199061 and PRJNA1198310. The whole-genome sequences of the MRSA ST9 and ST398 strains used in this study are available in the GenBank database, with accession numbers CP065194, CP031838, CP172432, and CP065199.

## SUPPLEMENTAL MATERIAL

Supplemental material msystems.00397-25-s0001.docx Supplemental figures and tables.

ASM does not own the copyrights to Supplemental Material that may be linked to, or accessed through, an article. The authors have granted ASM a non-exclusive, world-wide license to publish the Supplemental Material files. Please contact the corresponding author directly for reuse.

## References

[1] Anjum MF, Marco-Jimenez F, Duncan D, Marín C, Smith RP, Evans SJ. 2019. Livestock-associated methicillin-resistant Staphylococcus aureus from animals and animal products in the UK. Front Microbiol 10:2136. doi:10.3389/fmicb.2019.02136 31572341 PMC6751287

[2] Wang Y, Zhang P, Wu J, Chen S, Jin Y, Long J, Duan G, Yang H. 2023. Transmission of livestock-associated methicillin-resistant Staphylococcus aureus between animals, environment, and humans in the farm. Environ Sci Pollut Res 30:86521–86539. doi:10.1007/s11356-023-28532-7 37418185

[3] Silva V, Araújo S, Monteiro A, Eira J, Pereira JE, Maltez L, Igrejas G, Lemsaddek TS, Poeta P. 2023. Staphylococcus aureus and MRSA in livestock: antimicrobial resistance and genetic lineages. Microorganisms 11:124. doi:10.3390/microorganisms11010124 36677414 PMC9865216

[4] Chuang Y-Y, Huang Y-C. 2015. Livestock-associated meticillin-resistant Staphylococcus aureus in Asia: an emerging issue? Int J Antimicrob Agents 45:334–340. doi:10.1016/j.ijantimicag.2014.12.007 25593014

[5] Laumay F, Benchetrit H, Corvaglia A-R, van der Mee-Marquet N, François P. 2021. The Staphylococcus aureus CC398 lineage: an evolution driven by the acquisition of prophages and other mobile genetic elements. Genes (Basel) 12:1752. doi:10.3390/genes12111752 34828356 PMC8623586

[6] Jiang N, Wyres KL, Li J, Feßler AT, Krüger H, Wang Y, Holt KE, Schwarz S, Wu C. 2021. Evolution and genomic insight into methicillin-resistant Staphylococcus aureus ST9 in China. J Antimicrob Chemother 76:1703–1711. doi:10.1093/jac/dkab106 33822977

[7] Ji X, Krüger H, Tao J, Wang Y, Feßler AT, Bai R, Wang S, Dong Y, Shen J, Wang Y, Schwarz S, Wu C. 2021. Comparative analysis of genomic characteristics, fitness and virulence of MRSA ST398 and ST9 isolated from China and Germany. Emerg Microbes Infect 10:1481–1494. doi:10.1080/22221751.2021.1951125 34210245 PMC8300935

[8] Laux C, Peschel A, Krismer B. 2019. Staphylococcus aureus colonization of the human nose and interaction with other microbiome members. Microbiol Spectr 7. doi:10.1128/microbiolspec.gpp3-0029-2018 PMC11590430 31004422

[9] Foster TJ, Geoghegan JA, Ganesh VK, Höök M. 2014. Adhesion, invasion and evasion: the many functions of the surface proteins of Staphylococcus aureus. Nat Rev Microbiol 12:49–62. doi:10.1038/nrmicro3161 24336184 PMC5708296

[10] Wong Fok Lung T, Chan LC, Prince A, Yeaman MR, Archer NK, Aman MJ, Proctor RA. 2022. Staphylococcus aureus adaptive evolution: recent insights on how immune evasion, immunometabolic subversion and host genetics impact vaccine development. Front Cell Infect Microbiol 12:1060810. doi:10.3389/fcimb.2022.1060810 36636720 PMC9831658

[11] Liu CM, Price LB, Hungate BA, Abraham AG, Larsen LA, Christensen K, Stegger M, Skov R, Andersen PS. 2015. Staphylococcus aureus and the ecology of the nasal microbiome. Sci Adv 1:e1400216. doi:10.1126/sciadv.1400216 26601194 PMC4640600

[12] Wos-Oxley ML, Plumeier I, von Eiff C, Taudien S, Platzer M, Vilchez-Vargas R, Becker K, Pieper DH. 2010. A poke into the diversity and associations within human anterior nare microbial communities. ISME J 4:839–851. doi:10.1038/ismej.2010.15 20182526

[13] Fetsch A, Roesler U, Kraushaar B, Friese A. 2016. Co-colonization and clonal diversity of methicillin-sensitive and methicillin-resistant Staphylococcus aureus in sows. Vet Microbiol 185:7–14. doi:10.1016/j.vetmic.2016.01.011 26931385

[14] Livak KJ, Schmittgen TD. 2001. Analysis of relative gene expression data using real-time quantitative PCR and the 2(-delta delta C(T)) method. Methods 25:402–408. doi:10.1006/meth.2001.1262 11846609

[15] Sun N, Chen Y, Zhang J, Cao J, Huang H, Wang J, Guo W, Li X. 2023. Identification and characterization of pancreatic infections in severe and critical acute pancreatitis patients using 16S rRNA gene next generation sequencing. Front Microbiol 14:1185216. doi:10.3389/fmicb.2023.1185216 37389346 PMC10303115

[16] Edgar RC. 2004. MUSCLE: multiple sequence alignment with high accuracy and high throughput. Nucleic Acids Res 32:1792–1797. doi:10.1093/nar/gkh340 15034147 PMC390337

[17] Chaves-Moreno D, Wos-Oxley ML, Jáuregui R, Medina E, Oxley APA, Pieper DH. 2015. Application of a novel “pan-genome”-based strategy for assigning rnaseq transcript reads to Staphylococcus aureus strains. PLoS One 10:e0145861. doi:10.1371/journal.pone.0145861 26717500 PMC4696825

[18] Piewngam P, Otto M. 2024. Staphylococcus aureus colonisation and strategies for decolonisation. Lancet Microbe 5:e606–e618. doi:10.1016/S2666-5247(24)00040-5 38518792 PMC11162333

[19] Reynaga E, Navarro M, Vilamala A, Roure P, Quintana M, Garcia-Nuñez M, Figueras R, Torres C, Lucchetti G, Sabrià M. 2016. Prevalence of colonization by methicillin-resistant Staphylococcus aureus ST398 in pigs and pig farm workers in an area of Catalonia, Spain. BMC Infect Dis 16:716. doi:10.1186/s12879-016-2050-9 27894267 PMC5127002

[20] Uhlemann A-C, Porcella SF, Trivedi S, Sullivan SB, Hafer C, Kennedy AD, Barbian KD, McCarthy AJ, Street C, Hirschberg DL, Lipkin WI, Lindsay JA, DeLeo FR, Lowy FD. 2012. Identification of a highly transmissible animal-independent Staphylococcus aureus ST398 clone with distinct genomic and cell adhesion properties. mBio 3:e00027-12. doi:10.1128/mBio.00027-12 22375071 PMC3302565

[21] Billings C, Rifkin R, Abouelkhair M, Jones RD, Bow A, Kolape J, Rajeev S, Kania S, Anderson DE. 2022. In vitro and in vivo assessment of caprine origin Staphylococcus aureus ST398 strain UTCVM1 as an osteomyelitis pathogen. Front Cell Infect Microbiol 12:1015655. doi:10.3389/fcimb.2022.1015655 36726643 PMC9885270

[22] Chen F, Yin Y, Chen H, Jin L, Li S, Wang R, Wang S, Wang Q, Sun S, Wang H. 2022. Phenotypic and genomic comparison of Staphylococcus aureus highlight virulence and host adaptation favoring the success of epidemic clones. mSystems 7:e00831-22. doi:10.1128/msystems.00831-22 PMC9765012 36409083

[23] Lu H, Zhao L, Si Y, Jian Y, Wang Y, Li T, Dai Y, Huang Q, Ma X, He L, Li M. 2021. The surge of hypervirulent ST398 MRSA lineage with higher biofilm-forming ability is a critical threat to clinics. Front Microbiol 12:636788. doi:10.3389/fmicb.2021.636788 33746929 PMC7969815

[24] Schlattmann A, von Lützau K, Kaspar U, Becker K. 2020. The porcine nasal microbiota with particular attention to livestock-associated methicillin-resistant Staphylococcus aureus in Germany-a culturomic approach. Microorganisms 8:514. doi:10.3390/microorganisms8040514 32260366 PMC7232296

[25] Hardy BL, Merrell DS. 2021. Friend or foe: interbacterial competition in the nasal cavity. J Bacteriol 203:e00480-20. doi:10.1128/JB.00480-20 33077632 PMC7890553

[26] Zeng Y, Liang JQ. 2022. Nasal microbiome and its interaction with the host in childhood asthma. Cells 11:3155. doi:10.3390/cells11193155 36231116 PMC9563732

[27] Sikorska H, Smoragiewicz W. 2013. Role of probiotics in the prevention and treatment of meticillin-resistant Staphylococcus aureus infections. Int J Antimicrob Agents 42:475–481. doi:10.1016/j.ijantimicag.2013.08.003 24071026

[28] Yao D, Wang X, Ma L, Wu M, Xu L, Yu Q, Zhang L, Zheng X. 2022. Impact of Weissella cibaria BYL4.2 and its supernatants on Penicillium chrysogenum metabolism. Front Microbiol 13. doi:10.3389/fmicb.2022.983613 PMC9581191 36274712

[29] Yeu J-E, Lee H-G, Park G-Y, Lee J, Kang M-S. 2021. Antimicrobial and antibiofilm activities of Weissella cibaria against pathogens of upper respiratory tract infections. Microorganisms 9:1181. doi:10.3390/microorganisms9061181 34070813 PMC8229644

[30] Sakr A, Brégeon F, Mège J-L, Rolain J-M, Blin O. 2018. Staphylococcus aureus nasal colonization: an update on mechanisms, epidemiology, risk factors, and subsequent infections. Front Microbiol 9:2419. doi:10.3389/fmicb.2018.02419 30349525 PMC6186810

[31] Adolf LA, Heilbronner S. 2022. Nutritional interactions between bacterial species colonising the human nasal cavity: current knowledge and future prospects. Metabolites 12:489. doi:10.3390/metabo12060489 35736422 PMC9229137

[32] Krismer B, Liebeke M, Janek D, Nega M, Rautenberg M, Hornig G, Unger C, Weidenmaier C, Lalk M, Peschel A. 2014. Nutrient limitation governs Staphylococcus aureus metabolism and niche adaptation in the human nose. PLoS Pathog 10:e1003862. doi:10.1371/journal.ppat.1003862 24453967 PMC3894218

[33] Raineri EJM, Altulea D, van Dijl JM. 2022. Staphylococcal trafficking and infection-from “nose to gut” and back. FEMS Microbiol Rev 46:fuab041. doi:10.1093/femsre/fuab041 34259843 PMC8767451

[34] Stephens AC, Banerjee SK, Richardson AR. 2023. Specialized phosphate transport is essential for Staphylococcus aureus nitric oxide resistance. mBio 14:e02451-23. doi:10.1128/mbio.02451-23 37937971 PMC10746193

[35] Borezée-Durant E, Hiron A, Piard J-C, Juillard V. 2009. Dual role of the oligopeptide permease Opp3 during growth of Staphylococcus aureus in milk. Appl Environ Microbiol 75:3355–3357. doi:10.1128/AEM.02819-08 19286789 PMC2681649

[36] Ha KP, Edwards AM. 2021. DNA repair in Staphylococcus aureus. Microbiol Mol Biol Rev 85:e00091-21. doi:10.1128/MMBR.00091-21 34523959 PMC8483670

[37] Krüger-Haker H, Ji X, Bartel A, Feßler AT, Hanke D, Jiang N, Tedin K, Maurischat S, Wang Y, Wu C, Schwarz S. 2022. Metabolic characteristics of porcine LA-MRSA CC398 and CC9 isolates from Germany and China via biolog phenotype MicroArray Microorganisms 10:2116. doi:10.3390/microorganisms10112116 36363707 PMC9693340

[38] Helekal D, Keeling M, Grad YH, Didelot X. 2023. Estimating the fitness cost and benefit of antimicrobial resistance from pathogen genomic data. J R Soc Interface 20:20230074. doi:10.1098/rsif.2023.0074 37312496 PMC10265023

[39] Zhao Q, Jiang Z, Li T, Cheng M, Sun H, Cui M, Zhang C, Xu S, Wang H, Wu C. 2023. Current status and trends in antimicrobial use in food animals in China, 2018–2020. One Health Adv 1. doi:10.1186/s44280-023-00029-5

[40] Zheng L, Jiang Z, Wang Z, Li Y, Jiao X, Li Q, Tang Y. 2023. The prevalence of Staphylococcus aureus and the emergence of livestock-associated MRSA CC398 in pig production in eastern China. Front Microbiol 14:1267885. doi:10.3389/fmicb.2023.1267885 38163065 PMC10755019

[41] Witte W, Strommenger B, Stanek C, Cuny C. 2007. Methicillin-resistant Staphylococcus aureus ST398 in humans and animals, Central Europe. Emerg Infect Dis 13:255–258. doi:10.3201/eid1302.060924 17479888 PMC2725865

