# Supplementary material for: Comparative analysis of colonization and survival strategies of regionally predominant LA-MRSA clones ST398 and ST9
Source: mSystems. 2025 Sep 9;10(10):e00397-25. doi: 10.1128/msystems.00397-25 (PMC12542691; doi:10.1128/msystems.00397-25)

**Comparative Analysis of Colonization and Survival Strategies of Regionally Predominant LA-MRSA Clones ST398 and ST9**

**Xing Ji^1,2^, Yaxin Wang^1^, Tao He^2^, Henrike Krüger-Haker^3,4^, Yang Wang^1^, Congming Wu^1^, Stefan Schwarz^1,3,4^ and Chengtao Sun^1^**

^1^National Key Laboratory of Veterinary Public Health and Safety, College of Veterinary Medicine, China Agricultural University, Beijing, China.

^2^Jiangsu Key Laboratory for Food Quality and Safety—State Key Laboratory Cultivation Base of Ministry of Science and Technology, Institute of Food Safety and Nutrition, Jiangsu Academy of Agricultural Sciences, Nanjing 210040, China

^3^Institute of Microbiology and Epizootics, School of Veterinary Medicine, Centre for Infection Medicine, Freie University Berlin, Berlin, Germany.

^4^Veterinary Centre of Resistance Research, School of Veterinary Medicine, Freie University Berlin, Berlin, Germany.

Xing Ji and Yaxin Wang contributed equally to this article.

Address correspondence to Chengtao Sun, sct@cau.edu.cn; Stefan Schwarz, stefan.schwarz@fu-berlin.de.

**Supplemental material**

**Table S1** Basic information of MRSA ST9 and MRSA ST398 strains.

**Table S2** Representative strains and selected antimicrobial resistances of MRSA ST398 and ST9 isolated from China and Germany.

**Table S3.** Analysis of α diversity correlation index of the structure of swine nasal flora in each group of pigs before implantation. (Analysis of Variance, ANOVA).

**Table S4.** Taxonomic groups with significant abundance changes following colonization by MRSA ST9 and ST398 strains.

**Figure S1. Phylogenetic analysis of SA ST398 and ST9 strains.** The core genome SNPs phylogenetic tree of ST398 (a) and ST9 strains (b).

**Figure S2. The genus composition of microbial communities in samples of each group before colonization of MRSA ST9 and ST398 strains.** The relative abundance of bacterial communities was quantified using transcripts per million (TPM).

**Figure S3. β-diversity analysis using the ANOSIM (Analysis of Similarities) index was conducted at 1, 3, 7, and 14 days post-colonization.** Results showing R > 0 and *P* < 0.05 indicate significant differences in the compositional structure of bacterial communities among the different groups.

**Figure S4. Comparison of different bacterial genera among different groups of colonized strains at day 1, 3,14, and 21.** The graph illustrates differences in bacterial abundance between the vertical and horizontal axis groups. Red dots represent bacterial genera with significantly increased relative abundance in the vertical axis group, while green dots indicate genera with significantly decreased relative abundance in this group.

**Figure S5 Composition distribution of active bacterial phyla in pig nasal samples of each group on the first day based on metatranscriptome sequencing.** The relative abundance of bacterial communities was quantified using transcripts per million (TPM).

**Figure S6. PCoA analysis based on Bray-Curtis distance reflects the expression and composition of functional genes in different sample groups. (**A) PCoA analysis based on COGs annotation of functional genes. (B) PCoA analysis based on KEGG pathway of functional genes.

**Table S1**

|  | CHN-MRSA  ST9 | GER-MRSA  ST9 | GER-MRSA ST398 | CHN-MRSA ST398 |
| --- | --- | --- | --- | --- |
|  | QDCD9 | DG36 | DG29 | YN523 |
| Genome size (bp) | 2,855,865 | 2,752,938 | 2,810,778 | 2,839,435 |
| GC content（%） | 32.8% | 32.8% | 32.9% | 32.8% |
| CDS | 2734 | 2741 | 2824 | 2921 |
| Genomic island |  |  |  |  |
| vSaα | *vwb, aadE, scn, ssl(11), lpl(9)* | *vwb, scn, ssl(11), lpl(9)* | *vwb, scn, ssl(9), lpl(9)* | *ssl(9), lpl(9)* |
| vSaβ | enterotoxin*(8)* | enterotoxin*(8)* | *hysA* | *hysA* |
| SCC*mec* |  |  |  |  |
| type XII | *mecA* | *-* | *-* | *-* |
| type IV | *-* | *mecA* | *-* | *-* |
| type Vc | *-* | *-* | *mecA* | *mecA* |
| Transposons |  |  |  |  |
| Tn*552* | *copB, blaZ* | *copB* | *-* | *blaZ* |
| Tn*554-like* | *blaZ* | *blaZ* | *vgaE, blaZ* | *spw, lsa*(E)*, lnu*(B) |
| Tn*7* | *-* | *-* | NI | NI |
| Tn*558* | *fexA* | *-* | *-* | *-* |
| Tn*916*-like | *-* | *-* | *tet*(M) | *tet*(M) |
| Other resistance gene cluster | *aadE-spw-lsa*(E)*-lnu*(B) | *-* | *-* | *-* |
| Other antibiotic resistance genes | *erm(C), dfrG, tet(L),aadD* | *str* | *tet(K), str* | *fexA, cfr, erm(C), dfrG，spc* |
|  |  |  |  |  |
| Genetic evolution branch | Clada b3 | Clada b2 | Clada a2 | Clada a2 |
| Antibiotic resistance phenotypes | Tetracycline  Gentamicin  Ciprofloxacin  Florfenicol  Clindamycin  Erythromycin  Tiamulin  Vancomycin M1 | Gentamicin, Clindamycin | Tetracycline  Minocycline  Clindamycin | Tetracycline  Minocycline  Gentamicin  Ciprofloxacin  Florfenicol  Clindamycin  Erythromycin  Tiamulin  Vancomycin M1 |

**Table S2**

| **Origin** | **GenBank** | **ID** | **Strain-specific resistant antibiotics and MIC values** |
| --- | --- | --- | --- |
| CHN MRSA ST398 | CP065194 | YN523 | tiamulin (32 μg/mL) and minocycline (2 μg/mL) |
| CHN MRSA ST9 | CP031838 | QDT9 | florfenicol (8 μg/mL) and tiamulin (32 μg/mL) |
| GER MRSA ST398 | CP172432 | DG29 | minocycline (2 μg/mL) |
| GER MRSA ST9 | CP065199 | DG36 | ciprofloxacin (4 μg/mL) |

**Table S3.**

| **Index** | **CHN**  **ST398** | **GER**  **ST398** | **CHN**  **ST9** | **GER**  **ST9** | **Control** | ***P* value** |
| --- | --- | --- | --- | --- | --- | --- |
| chao1 | 1207.7 | 621.3 | 1464.7 | 1325.2 | 1197.9 | 0.3704 |
|  | 1453 | 1048.3 | 1419.4 | 1218.6 | 1220.6 |  |
|  | 985 | 1344.7 | 1269.8 | 957 | 1374 |  |
| shannon_2 | 4.86 | 4.28 | 6.74 | 6.74 | 4.87 | 0.4772 |
|  | 6.76 | 3.85 | 5.78 | 5.27 | 4.73 |  |
|  | 3.79 | 4.33 | 5.39 | 3.52 | 7.22 |  |
| simpson | 0.12 | 0.202 | 0.0358 | 0.0297 | 0.117 | 0.2599 |
|  | 0.0407 | 0.13 | 0.0571 | 0.129 | 0.137 |  |
|  | 0.164 | 0.249 | 0.0727 | 0.274 | 0.0174 |  |

**Table S4.**

| **Matched Groups** | **Significantly increased**  **genus (phylum)** | **Significantly reduced**  **genus (phylum)** |
| --- | --- | --- |
| CHN ST398  (GER ST398) | *Clostridium (Firmicutes)* | *Comamonas (Proteobacteria)*  *Chryseobacterium (Bacteroidota)*  *Kurthia (Firmicutes)* |
| CHN ST398  (CHN ST9) | *Acinetobacter (Proteobacteria)*  *Sphingomonas (Proteobacteria)*  *Roseburia (Firmicutes)*  *Empedobacter (Bacteroidota)* | *Faecalibacterium (Firmicutes)*  *Aerococcus (Firmicutes)* |
| GER ST398  (CHN ST9) | *Comamonas (Proteobacteria)*  *Sphingobacterium (Bacteroidota)*  *Aeromonas (Proteobacteria)*  *Arthrobacter (Actinobacteria)*  *Kurthia (Firmicutes)*  *Pseudomonas (Proteobacteria)* | *Weissella (Firmicutes)*  *Pediococcus (Firmicutes)*  *Faecalibacterium (Firmicutes)* |
| GER ST398  (GER ST9) | *Arthrobacter (Actinomycetota)*  *Brevundimonas (Pseudomonadota)*  *Dietzia (Actinomycetota)*  *Comamonas (Pseudomonadota)*  *Pseudomonas (Pseudomonadota)*  *Empedobacter (Bacteroidota)* | *Faecalibacterium (Firmicutes)* |
| GER ST9  (CHN ST9) | *Micrococcus (Actinobacteria)*  *Planococcus (Firmicutes)*  *Weissella (Firmicutes)* | *Pseudoramibacter (Firmicutes)* |

Note: Species with significantly increased or decreased abundance in each group (control group in parentheses), these genera consistently showed changes in abundance at least at two sampling times. Screening criteria were: *P*-value < 0.05 and log₂ Fold Change ≥ 2.

**Figure S1.**

**
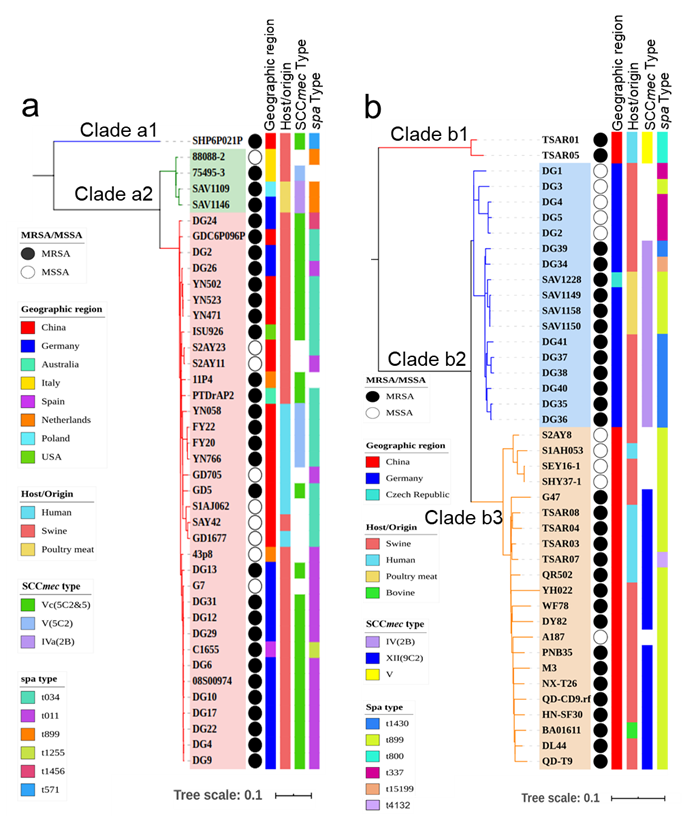
**Note: The figure is quoted from the author's previous research results (Ji Xing et al., *Emerging microbes & infections*. 2021).

**Figure S2.**


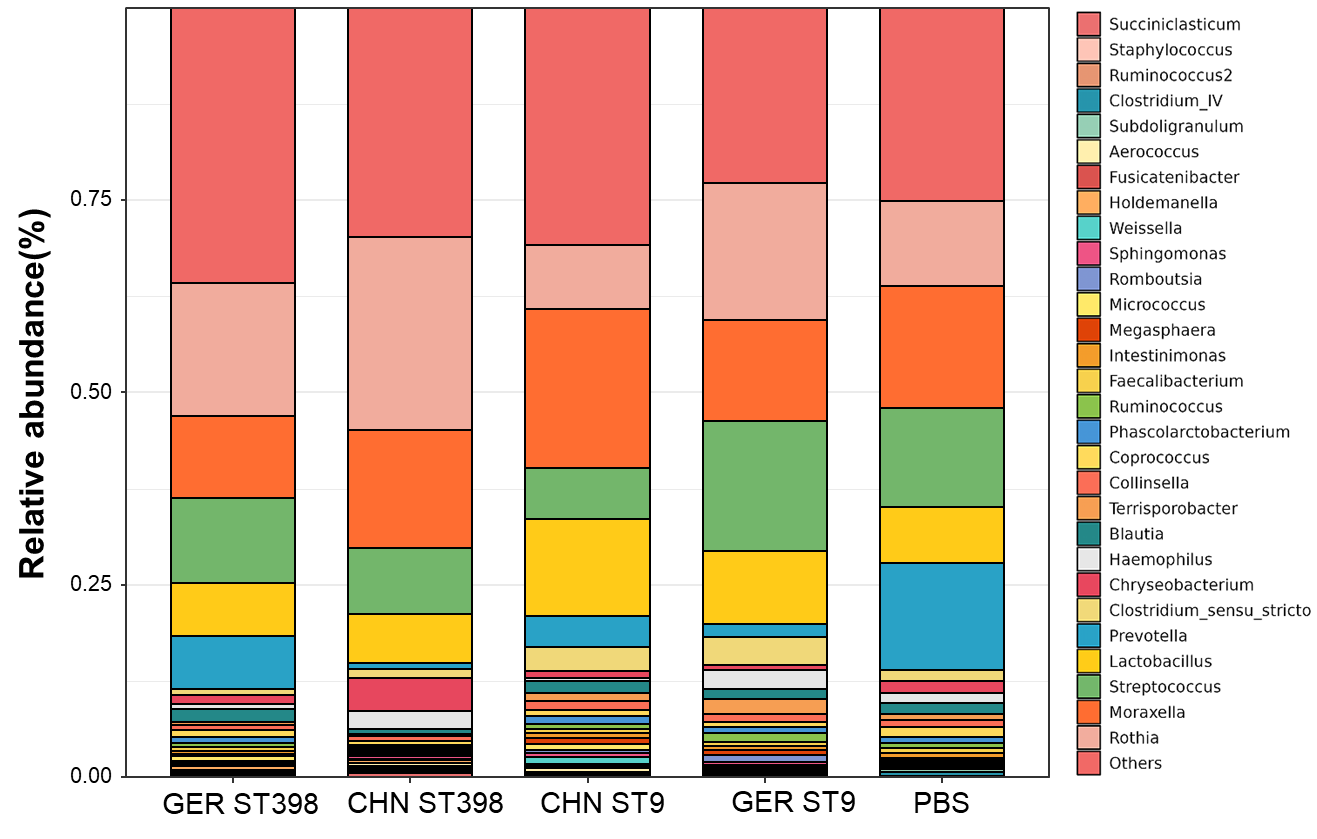


**Figure S3.**

**
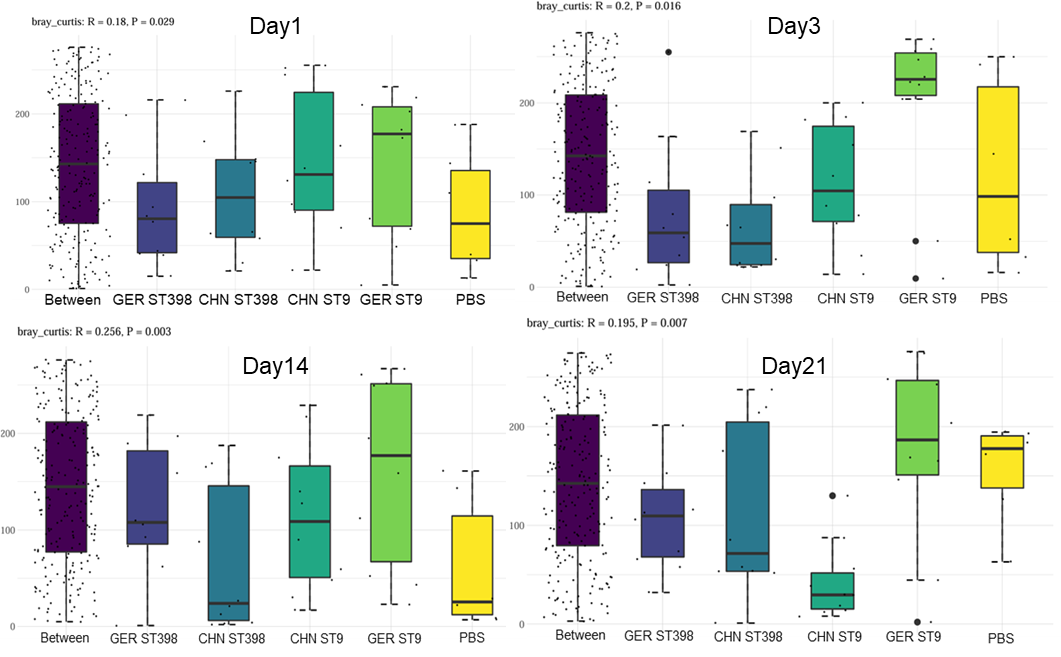
**

**
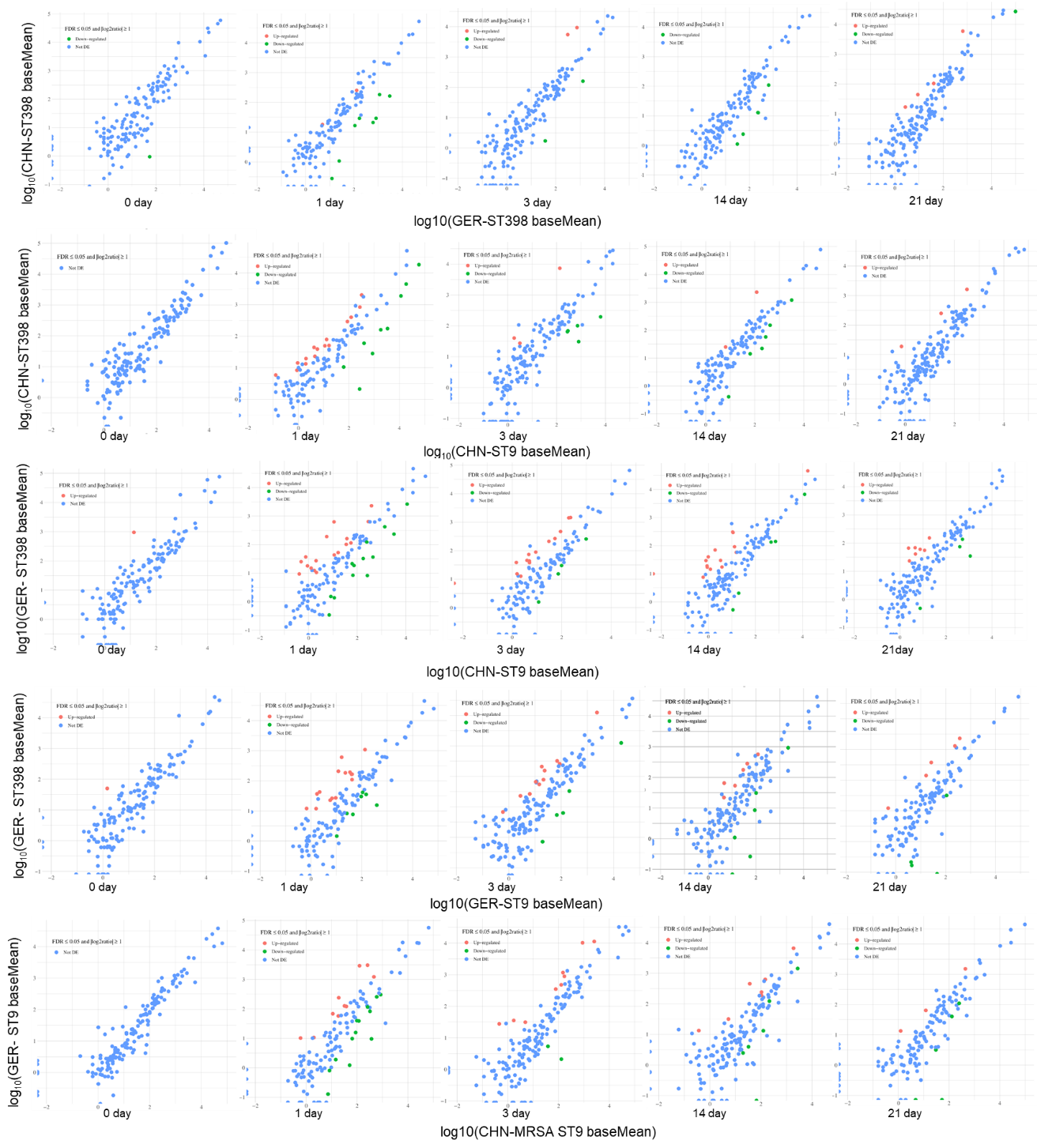
Figure S4.**

**Figure S5.**


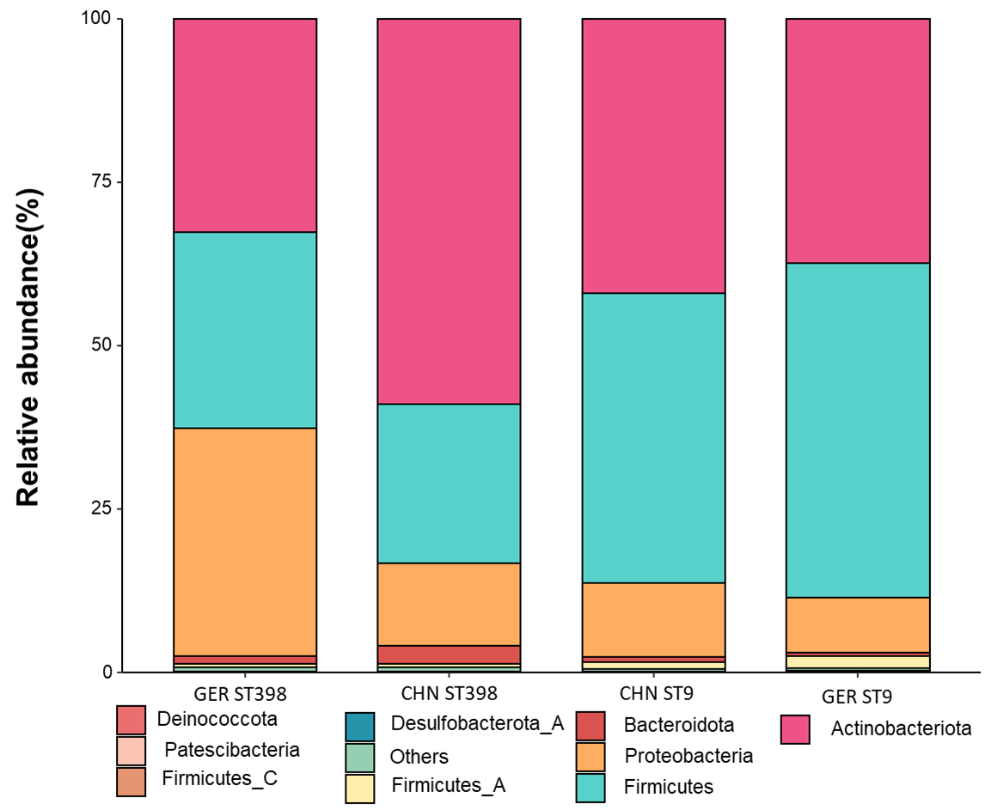


**Figure S6.**
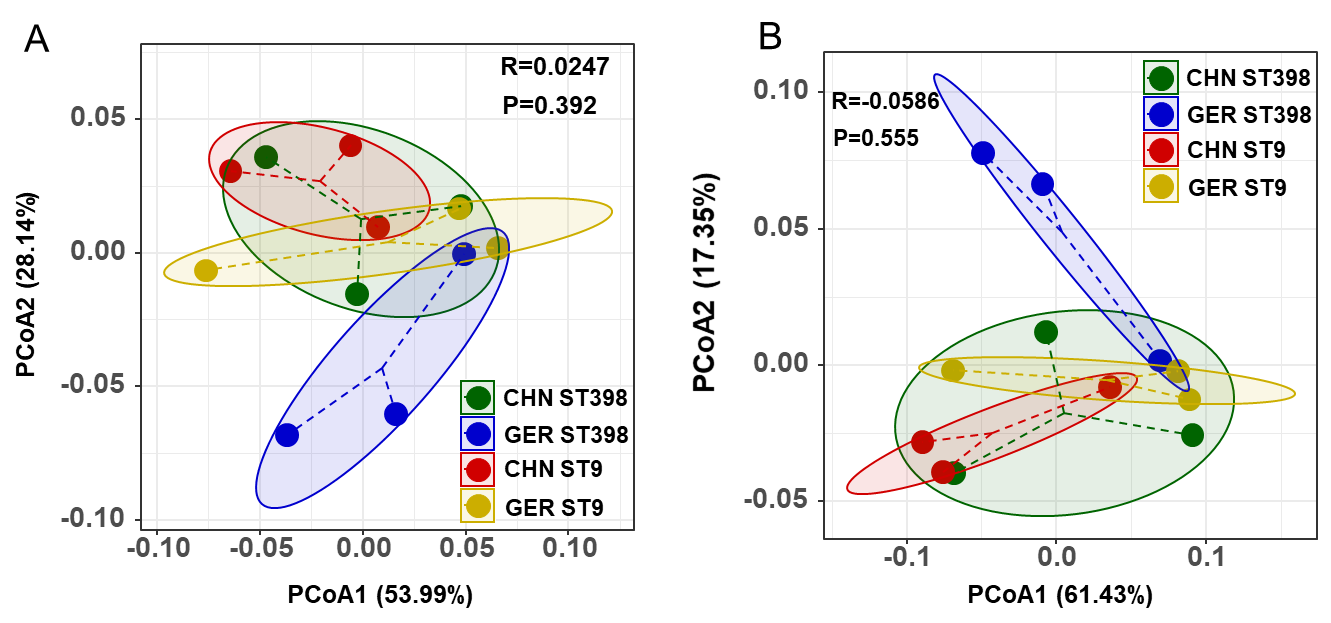

Supplement: Supplemental material — Supplemental figures and tables. [file msystems.00397-25-s0001.docx]
